# Thyroid dysfunction in patients with chronic urticaria

**DOI:** 10.1186/1939-4551-8-S1-A237

**Published:** 2015-04-08

**Authors:** Rita De Cássia De Souza Lopes, Rosana Câmara Agondi, Antônio Abílio Motta

**Affiliations:** 1 Hospital Das Clínicas Da University of São Paulo, Brazil

## Background

In chronic urticaria, thyroid dysfunction is not an uncommon association. Hypothyroidism and, less commonly, hyperthyroidism, may be associated with chronic spontaneous urticaria. The aim of this study was to evaluate the presence of thyroid dysfunction in patients with chronic urticaria followed in a tertiary outpatient clinic.

## Methods

A retrospective study of medical records evaluated 37 outpatients with chronic urticaria (CU), which means persistent symptoms lasting more than 6 weeks. Patients of both genders and aged over 18 years were included. Patients were classified according to the chronic urticaria in spontaneous, physical or autoimmune (positive autologous skin test). They were evaluated for the presence of any thyroid dysfunction or presence of antithyroid autoantibodies. We also evaluated the time of urticaria and age at diagnosis of thyroid dysfunction.

## Results

Thirty-seven patients with CU and thyroid dysfunction were included. Of these, 32 were female (86.5%), the mean age at study outset was 54.8 years and the mean duration of CU was 10.5 years. Of the 37 patients, 29 had spontaneous CU, 4 had autoimmune CU, 3 patients had CU dermographic, and 1 patient had delayed pressure urticaria. Eighteen patients (48.6%) presented hypothyroidism and five patients (13.5%), hyperthyroidism. Four patients had thyroid cancer and 7 had only positive antithyroid autoantibodies. The onset of the thyroid dysfunction occurs after CU in 79% of patients, with a time interval between 2 and 44 years (mean 15.2 years).

## Conclusions

The association of CU and thyroid dysfunction is highly prevalent, some studies showed values ranging from 6.5% to 57%. In this study, the assessment of thyroid dysfunction in patients CU showed that the most common disease was hypothyroidism, the prevalence of female gender was too high and the majority of the patients had thyroid dysfunction after the diagnosis of CU.

